# Regulation of organic acid and hydrogen production by NADH/NAD^+^ ratio in *Synechocystis* sp. PCC 6803

**DOI:** 10.3389/fmicb.2023.1332449

**Published:** 2024-01-05

**Authors:** Minori Akiyama, Takashi Osanai

**Affiliations:** School of Agriculture, Meiji University, Kawasaki, Kanagawa, Japan

**Keywords:** Cyanobacteria, fermentation, hydrogen, organic acids, succinate, lactate, acetate

## Abstract

Cyanobacteria serve as useful hosts in the production of substances to support a low-carbon society. Specifically, the unicellular cyanobacterium *Synechocystis* sp. PCC 6803 (*Synechocystis* 6803) can produce organic acids, such as acetate, lactate, and succinate, as well as hydrogen, under dark, anaerobic conditions. The efficient production of these compounds appears to be closely linked to the regulation of intracellular redox balance. Notably, alterations in intracellular redox balance have been believed to influence the production of organic acids and hydrogen. To achieve these alterations, genetic manipulations involved overexpressing malate dehydrogenase (MDH), knocking out d-lactate dehydrogenase (DDH), or knocking out acetate kinase (AK), which subsequently modified the quantities and ratios of organic acids and hydrogen under dark, anaerobic conditions. Furthermore, the mutants generated displayed changes in the oxidation of reducing powers and the nicotinamide adenine dinucleotide hydrogen (NADH)/NAD^+^ ratio when compared to the parental wild-type strain. These findings strongly suggest that intracellular redox balance, especially the NADH/NAD^+^ ratio, plays a pivotal role in the production of organic acids and hydrogen in *Synechocystis* 6803.

## 1 Introduction

In recent years, the issue of fossil fuel depletion and climate change has gained prominence due to global population growth (Stephens et al., 2010; Baicha et al., 2016). The use of fossil fuels for energy and as raw materials in the chemical industry releases greenhouse gasses such as carbon dioxide (CO_2_), making it a significant contributor to climate change, including phenomena like global warming and ocean acidification (Baicha et al., 2016). Studies predict that if the world’s energy demand relies solely on fossil fuels, these finite reserves will be exhausted between 2069 and 2088 (Stephens et al., 2010). The low-carbon technologies are essential for transitioning to a carbon-neutral society and economy (Yamane and Osanai, 2023). Cyanobacteria, photosynthetic bacteria capable of producing various metabolites from CO_2_, have attracted attention as ideal hosts for these challenges (Oliver et al., 2016; Katayama et al., 2018; Yamane and Osanai, 2023).

*Synechocystis* sp. PCC 6803, referred to as *Synechocystis* 6803 hereafter, is a non-nitrogen-fixing unicellular cyanobacterium. It is widely employed as a model organism in both basic and applied studies (Vijay et al., 2019) due to its whole genomic information (Kaneko et al., 1996), natural transformation capability (Yu et al., 2013), and resilience to cryopreservation. *Synechocystis* 6803 fixes carbon dioxide via oxygenic photosynthesis and stores glycogen when subjected to nitrogen starvation (Neumann et al., 2021). Under dark, anaerobic conditions, glycogen is metabolized via the glycogen catabolism pathway, leading to the excretion of organic acids, including acetate, lactate, and the four-carbon dicarboxylic acids, including succinate, fumarate, and malate (Stal and Moezelaar, 1997; Osanai et al., 2015; Hasunuma et al., 2016). Notably, succinate, fumarate, and malate are listed in the twelve most important building block chemicals in biorefinery, as recognized by the U.S. Department of Energy (Werpy and Petersen, 2004). These compounds find universal applications in the food, pharmaceutical, and industrial sectors (Chen and Nielsen, 2016; Dai et al., 2018; Shi et al., 2022). Additionally, *Synechocystis* 6803 exhibits the ability to produce hydrogen under dark, anaerobic conditions, along with organic acids (Stal and Moezelaar, 1997; Tamagnini et al., 2007). Hydrogen is considered a promising energy resource due to its remarkable energy yield per unit mass, which surpasses hydrocarbon fuels by a factor of 2.75, and its emission of zero greenhouse gasses (Levin et al., 2004; Kapdan and Kargi, 2006). As a result, the diverse range of fermentation metabolites produced by *Synechocystis* 6803 has the potential to substitute for petroleum-derived substances, contributing significantly to the realization of a carbon-neutral society.

**Figure 1:**
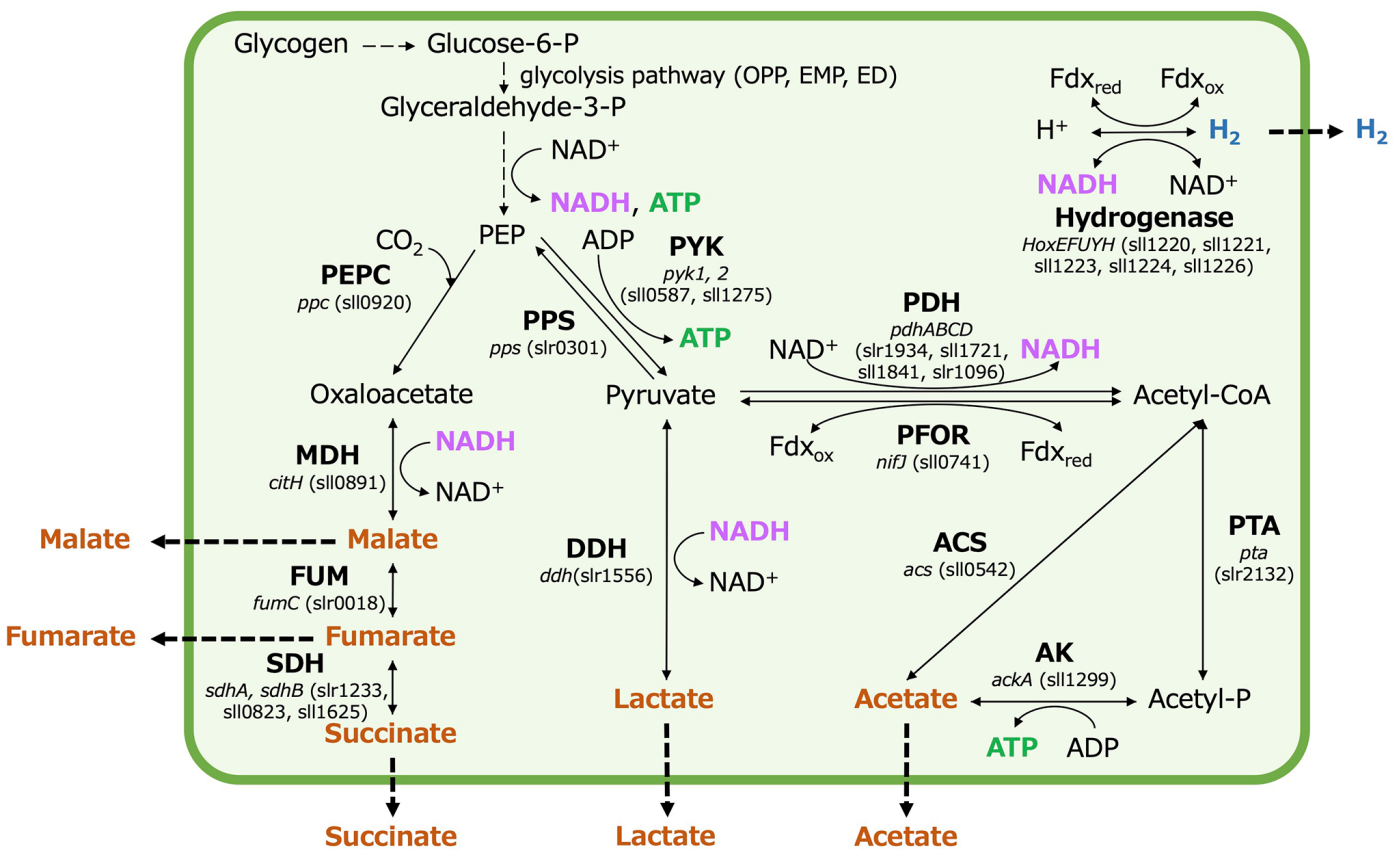
Production of fermentation metabolites in *Synechocystis* 6803 under dark, anaerobic conditions, with a focus on organic acid and hydrogen, nicotinamide adenine dinucleotide hydrogen (NADH), ferredoxin, and adenosine triphosphate (ATP) production. P designates phosphate. OPP; oxidative pentose phosphate, EMP; Embden–Meyerhof–Parnas, ED; the Entner–Doudoroff, PEP; phosphoenolpyruvate, PEPC; phosphoenolpyruvate carboxylase, PPS; phosphoenolpyruvate synthase, PYK; pyruvate kinase, MDH; malate dehydrogenase, FUM; fumarase, SDH; succinate dehydrogenase, DDH; d-lactate dehydrogenase, PDH; pyruvate dehydrogenase, PFOR; pyruvate: ferredoxin oxidoreductase, Fdx_red_; reduced ferredoxin, Fdx_ox_; oxidized ferredoxin, ACS; acetyl-CoA synthetase, PTA; phosphotransacetylase, and AK; acetate kinase.

Organic acids and hydrogen production by *Synechocystis* 6803, while promising, has not yet reached industrial-scale application (Dai et al., 2018; Liu et al., 2022; Opel et al., 2023). Under dark, anaerobic conditions, in *Synechocystis* 6803, acetate is produced by acetate kinase (*ackA*) (sll1299), lactate by d-lactate dehydrogenase (*ddh*) (slr1556), and four-carbon dicarboxylic acids by malate dehydrogenase (MDH) (encoded by *citH*) (sll0891), fumarase (*fumC*) (slr0018), and succinate dehydrogenase (*sdhA, sdhB*) (slr1233, sll0823, sll1625) (as illustrated in Figure 1). Acetate, lactate, and four-carbon dicarboxylic acids compete for carbon intermediates (phosphoenolpyruvate and pyruvate) between pathways (Osanai et al., 2015; Hasunuma et al., 2016; Iijima et al., 2021). Extensive research efforts have been undertaken to improve the production rate, yield, and titer of lactate and four-carbon dicarboxylic acids in terms of carbon intermediate competition. This involves the regulation of specific gene expression and the engineering of metabolic pathways (Khanna and Lindblad, 2015; Oliver et al., 2016; Kamshybayeva et al., 2022; Yamane and Osanai, 2023). Notably, when acetate kinase (AK) is lacking in the *ΔackA* strain, acetate production decreases while lactate and succinate production increases compared to the parental wild-type strain (Osanai et al., 2015). Conversely, overexpressing MDH, which catalyzes the conversion of oxaloacetate to malate, increases the production of four-carbon dicarboxylic acids while reducing acetate and lactate production (Iijima et al., 2021). On the other hand, in *Δddh* lacking lactate dehydrogenase (DDH), the production of succinate and lactate is lower than in the parental wild-type strain (Osanai et al., 2015). Additionally, in *Δacs* lacking acetyl-CoA synthetase (encoded by *acs*) (sll0542), which interconverts acetyl-CoA and acetate, the production of each organic acid decreases compared to the parental wild-type strain. However, the relative proportions of the organic acids remain similar to those in the parental wild-type strain (Osanai et al., 2015). Furthermore, the overexpression of phosphoenolpyruvate carboxylase (PEPC) (encoded by *ppc*) (sll0920), responsible for generating oxaloacetate from phosphoenolpyruvate (PEP) and bicarbonate ions, increases acetate and succinate production but does not significantly affect lactate production (Hasunuma et al., 2016). In addition to the regulation of the expression of genes related to organic acids metabolism, the pool size and turnover of metabolites have been analyzed by metabolomic analysis using a stable isotope such as ^13^C, and understanding of carbon source competition is steadily advancing (Hasunuma et al., 2018). However, it is important to note that while competition for carbon sources or intermediates plays an important role in organic acids production under dark, anaerobic conditions, it is also regulated by many other factors beyond carbon competition.

Hydrogen production by *Synechocystis* 6803 is catalyzed by a bidirectional hydrogenase (Carrieri et al., 2011), which can utilize NADH, reduced ferredoxin, and flavodoxin as substrates (Gutekunst et al., 2014). This hydrogenase plays a pivotal role in regulating the redox balance under dark, anaerobic conditions (Appel et al., 2000; Pinto et al., 2012). Additionally, when DDH and MDH in *Synechocystis* 6803 function as part of the fermentation pathway, they also utilize NADH (Angermayr et al., 2016; Takeya et al., 2018). Alterations in the expression of *ddh*, *citH*, and *hoxH* result in changes in NADH utilization, leading to either an increase or decrease in hydrogen production under dark, anaerobic conditions (Chongsuksantikul et al., 2015; Iijima et al., 2016, 2021). A flexible balance of ATP and NAD(P)H is crucial for metabolite production in *Synechocystis* 6803, whether in photoautotrophic or mixotrophic conditions (Kugler and Stensjö, 2023). In addition, lowering the adenylate energy charge has proven effective for succinate production under dark, anaerobic conditions in *Synechocystis* 6803 (Hasunuma et al., 2018). It is worth noting that the NADPH pool of *Synechocystis* 6803 is larger than the NADH pool (Takahashi et al., 2008), making the use of NADPH-dependent enzymes advantageous (Kugler and Stensjö, 2023). However, when transitioning to dark, anaerobic conditions, intracellular NADPH is entirely depleted (Osanai et al., 2015). The oxidative utilization of NADH by DDH, MDH, and hydrogenases is regulated at the gene expression level in response to environmental conditions, primarily due to differences in energy gain, substrate availability, and product formation within each fermentation pathway (Kariyazono and Osanai, 2023). Therefore, the intracellular redox balance of *Synechocystis* 6803 under dark, anaerobic conditions may be regulated differently compared to photoautotrophic and mixotrophic conditions. Such intracellular redox balance is expected to involve DDH, MDH, and hydrogenase, all of which use reducing power as substrates, as well as AK, which is crucial for ATP generation. Because of this complexity, there has been a notable absence of comprehensive discussions regarding carbon sources, the utilization of reducing power, intracellular ATP, or redox balance in metabolite production under dark anaerobic conditions. Furthermore, there is the technical difficulty of understanding all of the metabolites inside and outside the cell.

In this study, we conducted an analysis to explore the relationship between fermentation metabolite, organic acids and hydrogen production, and intracellular redox balance under dark, anaerobic conditions. We utilized various mutant strains of *Synechocystis* 6803 that had previously been genetically modified to increase organic acids and hydrogen production.

## 2 Materials and methods

### 2.1 Cyanobacterial strains and culture conditions

The glucose-tolerant (GT) strain of *Synechocystis* sp. PCC 6803, originally isolated by Williams (1988) and re-sequenced by Kanesaki et al. (2012), was cultivated in modified BG-11 medium, which consists of BG-11_0_ liquid medium (Rippka, 1988), supplemented with 5 mM NH_4_Cl (buffered with 20 mM HEPES-KOH, pH 7.8). Several mutant strains, including *Δddh* (lacking lactate dehydrogenase), *Δacs* (lacking acetyl-CoA synthetase), *ΔackA* (lacking acetate kinase) (Osanai et al., 2015), CitHox, (overexpressing *citH*) (Iijima et al., 2021), *ΔcitH* (lacking malate dehydrogenase) were previously generated (Katayama et al., 2022). Cyanobacterial cultures were aerated with air containing 1% (v/v) CO_2_ and incubated at 30°C under continuous white light (~50–60 μmol photons m^−2^ s^−1^) at the 70 mL scale. The *Δddh*, *Δacs*, *ΔackA*, and *ΔcitH* strains were pre-cultivated in the presence of 10 μg/mL chloramphenicol, while the CitHox strain was pre-cultivated with 10 μg/mL kanamycin. Cell densities were assessed at a wavelength of 730 nm wavelength (OD_730_) using a Shimadzu UV-2700 spectrophotometer (Shimadzu, Kyoto, Japan).

### 2.2 Plasmid construction

The CitHox strain was constructed by the following procedure by Iijima et al. (2021). The nucleotides corresponding to open reading frame of *Synechocystis* 6803 *citH* (sll0891) with an N–terminal *Nde*I site and a C-terminal *Eco*RV site was synthesized at Eurofin Genomics Japan (Tokyo, Japan). The synthetic DNA was digested using *Nde*I and *Eco*RV; the fragment was cloned into the *Nde*I–*Hpa*I sites of the pTKP2031 vector constructed by Satoh et al. (2001). The *citH* gene with the *psbAII* promoter (spanning −297 to +3 from the initiation codon of *psbAII*) was introduced into the neutral site within the open reading frame slr2031 with a kanamycin resistance in the *Synechocystis* 6803 genome by homologous recombination (Osanai et al., 2011). The plasmids were transformed into cells by natural transformation. Cell cultures (approximately 3 mL) were suspended to 100 μL, and 1 μL of plasmid solution (100 μg/mL) was added. The cell cultures were spread onto a mixed cellulose membrane on a BG-11 plate solidified with 1.5% agar and 2 mM sodium thiosulfate. After incubation at 30°C under continuous illumination (50 μmol photons m^−2^ s^−1^) overnight, the filter was transferred onto a BG-11 plate with 50 μg/mL kanamycin followed by incubation at 30°C under continuous illumination (50 μmol photons m^−2^ s^−1^) for 2–3 weeks. Colonies were re-streaked twice onto fresh BG-11 medium with kanamycin, and the strain overexpressing *citH* was designated as CitHox.

### 2.3 Dark and anaerobic incubation

Cells, initially cultured in 70 mL of modified BG-11 medium (started from OD_730_ = 0.4) for five days, were collected by centrifugation at 5800 × *g* for 2 min. Following the removal of the supernatant, the cells were concentrated in a 10 mL of a HEPES buffer (20 mM HEPES-KOH, pH 7.8) at OD_730_ = 20. This concentrated cell suspension was placed in a 20 mL GC-vial. The vials were flushed with N_2_ gas for 1 min using syringes to remove the oxygen and immediately sealed with butyl rubber and wrapped with aluminum foil. Subsequently, the vials were shaken at 30°C for three days. After this incubation period, the cell cultures underwent another round of centrifugation at 5800 × *g* for 2 min, following which the supernatant was filtered. A volume of 1 mL of the filtered supernatant was subjected to freeze-drying. The resulting dried supernatants were then utilized for the analysis of organic acids using high-performance liquid chromatography (HPLC).

### 2.4 Glycogen measurement

Equal volumes of cells (10 mL of cell culture with OD_730_ = 1.0) were collected through centrifugation (20,500 × *g* at 25°C for 1 min), with subsequent removal of the supernatant. The resulting cell pellets were then resuspended in 100 μL of 3.5% (w/v) sulfuric acid and subjected to incubation at 100°C for 80 min. Following another round of centrifugation (20,500 × *g* at 4°C for 1 min), 1.3 μL of the supernatant was combined with 200 μL of LabAssay Glucose reaction mixture (Fujifilm Wako Chemicals, Osaka, Japan) within a 96-well plate and incubated at 37°C for 15 min, and subsequently, the absorbance was measured at 595 nm using a Multiskan FC microplate reader (Thermo Scientific, MA, United States).

### 2.5 Measurement of excreted organic acids via high-performance liquid chromatography (HPLC)

The freeze-dried supernatants were reconstituted by dissolving in 100 μL of filtered 3 mM perchloric acid and analyzed by HPLC using an LC-2000Plus system (JASCO, Tokyo, Japan) equipped with two RSpak KC-811 columns (Showa Denko, Tokyo, Japan). Quantification of organic acids was achieved using a solution containing 0.2 mM bromothymol blue in 15 mM sodium phosphate buffer, with peak detection occurring at 445 nm. The column was maintained at a temperature of 60°C, and the flow rates were 0.7 mL/min for the 9 mM perchloric acid and 1.2 mL/min for the 0.2 mM bromothymol blue solution. To calibrate the measurements, standard powders of succinate, malate, fumarate, lactate, and acetate were employed, all of which were obtained from Fujifilm Wako Chemicals. Citrate powders were purchased from Nacalai Tesque, INC. (Kyoto, Japan).

### 2.6 Measurement of hydrogen evolution by gas chromatography-thermal conductivity detector (GC-TCD)

The cells were subjected to incubation under dark, anaerobic conditions, similar to the conditions used in the organic acids excretion experiment. The H_2_ gas that accumulated in the headspace of a GC-vial was quantified using a gas chromatograph (GC-2014 AT, Shimadzu, Kyoto, Japan). N_2_ was employed as the carrier gas, flowing at a flow rate of 10 mL/min.

### 2.7 Measurement of NADH and total NAD and NADH

Equal volumes of cells (1 mL of cell culture with OD_730_ = 50) were collected via centrifugation (300 × *g* at 25°C for 5 min) after the incubation period under dark, anaerobic conditions. The extraction and quantification of NAD^+^ from *Synechocystis* 6803 cells was carried out using the NAD/NADH Assay Kit-WST (Dojindo Laboratories, Kumamoto, Japan). The absorbance was measured at 450 nm using a Multiskan FC microplate reader (Thermo Scientific). To account for any background effects in visually colored samples, the absorbance before the reaction with the chromogenic dye mixture and the absorbance of the blank were considered.

### 2.8 Statistical analysis

The calculation of means and standard deviations, along with the determination of *p*-values, were conducted using Microsoft Excel 2019 MSO. All results were obtained through biologically independent replicates.

## 3 Result

### 3.1 Acetate kinase knockout increased the rate of glycogen consumption

**Figure 2:**
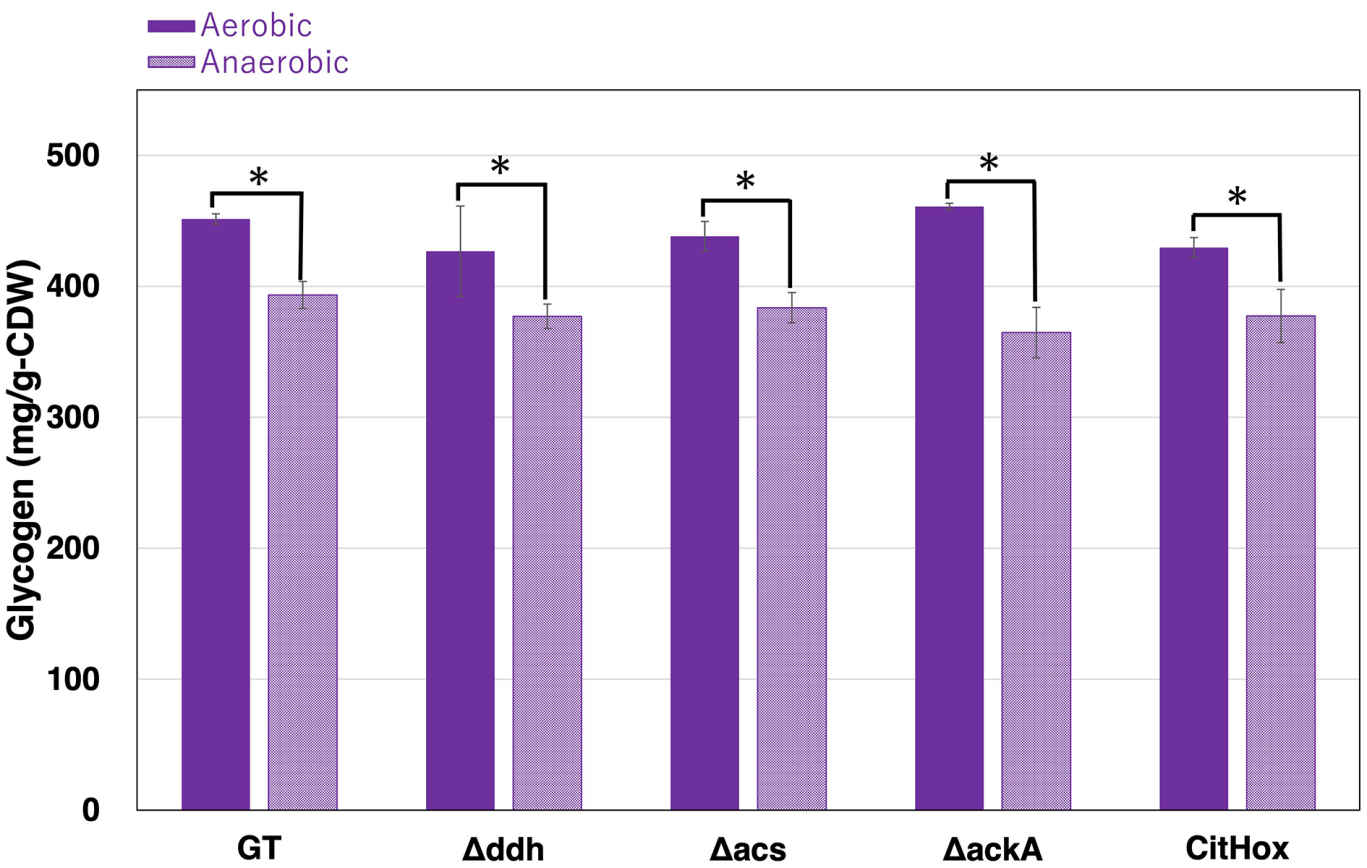
Changes in glycogen levels before and after dark, anaerobic conditions. *Synechocystis* 6803 GT, *Δddh*, *Δacs*, *ΔackA*, and CitHox were subjected to aerobic conditions with continuous illumination (50 μmol photons m^−2^ s^−1^) for five days. Following this, cells were incubated under dark, anaerobic conditions for three days. Glycogen levels were quantified using the LabAssay Glucose kit. The term “Anaerobic” indicates glycogen levels of the cells after three days of incubation under dark, anaerobic conditions. Data represents the means ± SD from four biologically independent experiments. Asterisks indicate statistically significant differences between glycogen levels before and after dark, anaerobic conditions for each strain (Student’s *t*-test; *p < 0.05).

Under continuous light and aerobic conditions, *Synechocystis* 6803 accumulates glycogen, utilizing it as a carbon and energy source under dark conditions (Stal and Moezelaar, 1997). After five days of light, aerobic cultivation, all strains, including GT, *Δddh*, *Δacs*, *ΔackA*, and CitHox, accounted similar glycogen levels for approximately 40–45% of cell dry weight (Figure 2). Subsequently, these cells were collected and concentrated in 10 mL of 20 mM HEPES buffer to OD_730_ = 20 before undergoing incubation under dark, anaerobic conditions for three days. Following this incubation, the glycogen levels in GT, *Δddh*, *Δacs*, and CitHox decreased by approximately 10%, and *ΔackA* decreased by approximately 20% compared to their initial glycogen levels before the dark, anaerobic incubation (Table 1).

**Table 1:** Relative glycogen levels before and after dark, anaerobic conditions.

Strain	Aerobic	Anaerobic
GT	100 ± 0.9	87.1 ± 2.3
*Δddh*	94.6 ± 7.6	83.5 ± 2.1
*Δacs*	97.1 ± 2.5	85.0 ± 2.5
*ΔackA*	102.1 ± 0.5	80.8 ± 4.3*
CitHox	95.2 ± 1.7	83.6 ± 4.5

Data represent means ± SD results from four independent experiments. Glycogen levels were calibrated relative to that of GT under light, aerobic conditions (set at 100%). Anaerobic indicates that glycogen levels of the cells incubated for three days under dark, anaerobic conditions. Asterisk indicates that statistically significant differences between GT and the mutant strain under the same conditions (Student’s t-test; *p < 0.05).

### 3.2 Knockout of *ddh*, *ackA*, or overexpression of *citH* altered the amount of organic acids production

**Figure 3:**
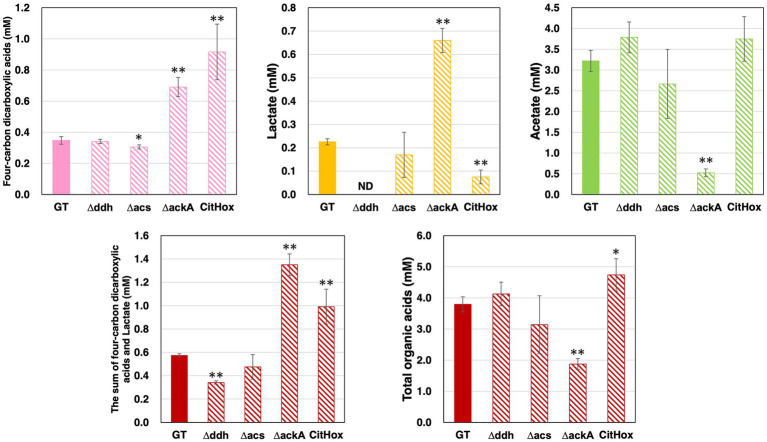
Quantification of four-carbon dicarboxylic acids, lactate, and acetate in *Synechocystis* 6803 GT, *Δddh*, *Δacs*, *ΔackA*, and CitHox under dark, anaerobic conditions. The levels of organic acids excreted from cells after three days of incubation under dark, anaerobic conditions were determined using high-performance liquid chromatography. Total organic acids represent the combined levels of four-carbon dicarboxylic acids, lactate, and acetate. Data is presented as means ± SD from three to four biologically independent experiments. Four-carbon dicarboxylic acids refer to the sum of succinate, fumarate, and malate. ND indicates undetectable levels. Asterisks indicate statistically significant differences between GT and the mutant strains (Student’s *t*-test; *p < 0.05, **p < 0.005).

After five days of growth of GT, *Δddh*, *Δacs*, *ΔackA*, and CitHox under light, aerobic conditions, followed by three days of incubation under dark, anaerobic conditions, the excreted organic acids were quantified. The sum of four-carbon dicarboxylic acids (succinate, fumarate, and malate) differed among strains, with GT producing 0.35 ± 0.02 mM, *ackA* knockout yielding 0.69 ± 0.06 mM, and *citH* overexpression resulting in 0.92 ± 0.18 mM under the same conditions (Figure 3). In contrast, *acs* knockout decreased the sum of four-carbon dicarboxylic acids levels by 12%, while *ddh* knockout did not significantly affect these levels (Figure 3). Lactate levels also varied among strains, with GT producing 0.23 ± 0.01 mM, *ackA* knockout yielding 0.66 ± 0.05 mM, and *citH* overexpression resulting in 0.08 ± 0.03 mM (Figure 3). The *ddh* knockout led to undetectable lactate production, and *acs* knockout did not affect lactate levels (Figure 3). Regarding acetate production, GT produced 3.22 ± 0.25 mM, and *ackA* knockout produced 0.52 ± 0.09 mM (Figure 3). No significant changes in acetate levels were observed with *citH* overexpression, *ddh* or *acs* knockout (Figure 3). The sum of four-carbon dicarboxylic acids and lactate decreased by 41% for *Δddh* and increased by 136% for *ΔackA* and by 73% for CitHox compared to GT (Figure 3). Overall, the total organic acids production, encompassing the sum of four-carbon dicarboxylic acids, lactate, and acetate, decreased by 51% for *ΔackA* and increased by 25% for CitHox compared to GT (Figure 3).

### 3.3 Knockout or overexpression of genes related to organic acids production altered hydrogen production

**Figure 4:**
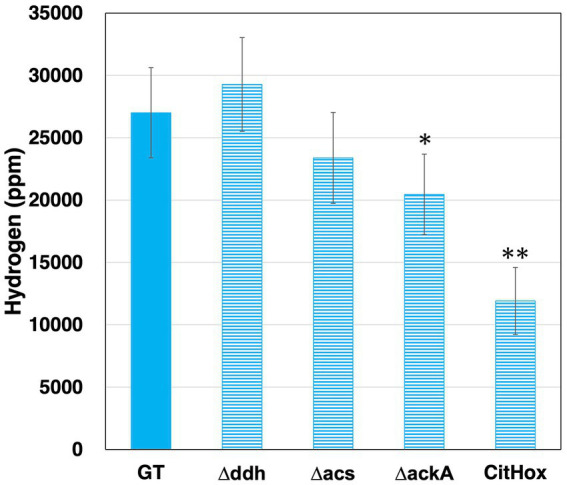
Quantification of hydrogen levels in *Synechocystis* 6803 GT, *Δddh*, *Δacs*, *ΔackA*, and CitHox cells under dark, anaerobic conditions. The concentration of hydrogen was determined using gas chromatography with a thermal conductivity detector. Data is presented as means ± SD from three to four biologically independent experiments. Asterisks indicate statistically significant differences between GT and the mutant strains (Student’s *t*-test; *p < 0.05, **p < 0.005).

*Synechocystis* 6803 produces hydrogen along with organic acids under dark anaerobic conditions (Stal and Moezelaar, 1997; Tamagnini et al., 2007). To examine the relationship between hydrogen and organic acid production, GT, *Δddh*, *Δacs*, *ΔackA*, CitHox, and *ΔcitH* were incubated under dark anaerobic conditions for 3 days, and hydrogen levels were quantified. GT produced hydrogen at a level of 27010 ± 3610 ppm, *ackA* knockout yielded 20463 ± 3228 ppm, and *citH* overexpression resulted in 11906 ± 2694 ppm under the same conditions (Figure 4). The *ddh* or *acs* knockout did not significantly affect hydrogen levels (Figure 4). In contrast, when *citH* was knocked out, hydrogen production increased by 137% to 36896 ± 2475 ppm compared to GT (Supplementary Figure S1a).

### 3.4 Knockout or overexpression of genes related to organic acids production altered intracellular NADH/NAD^+^ ratios

**Figure 5:**
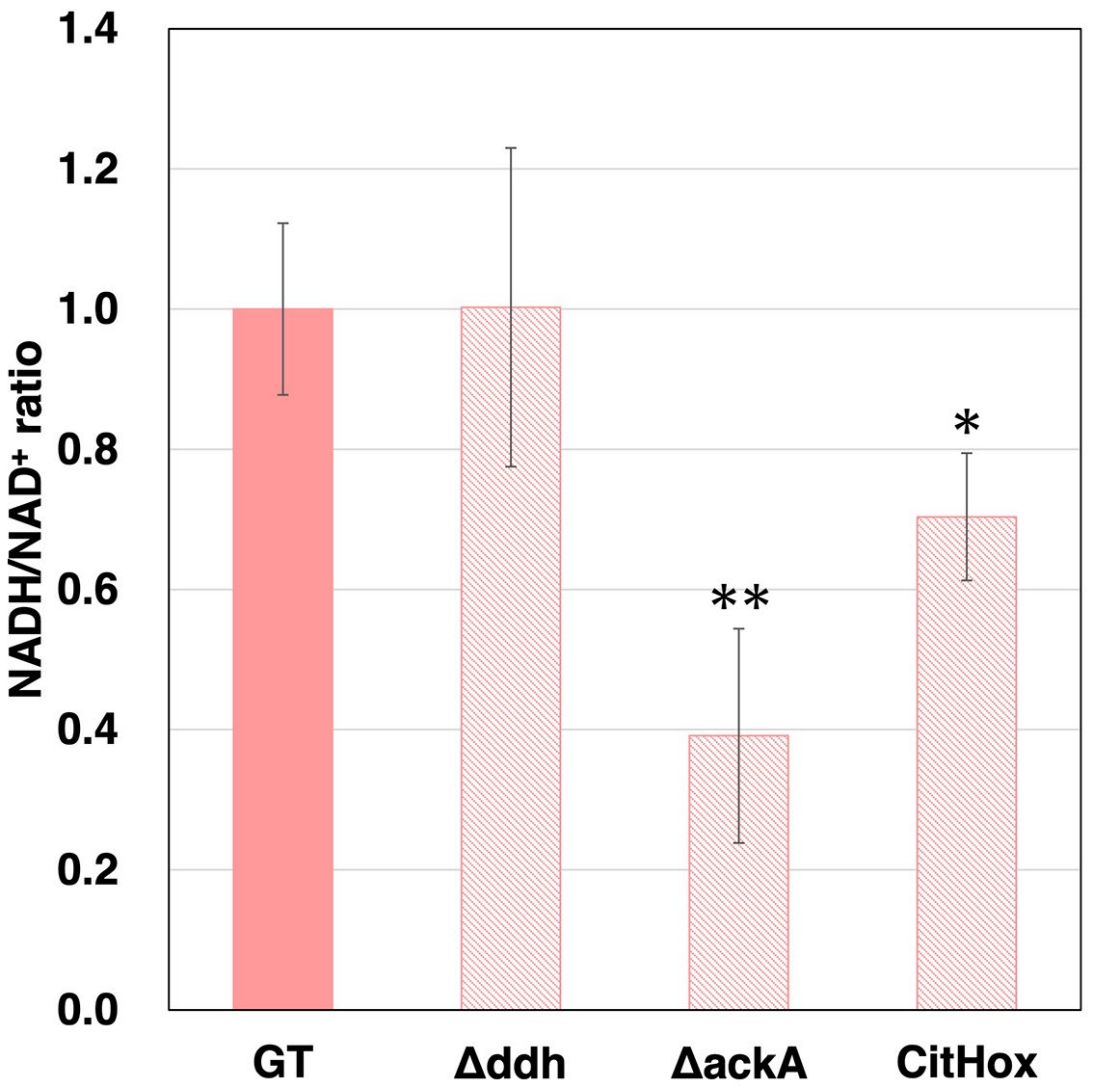
Intracellular NADH/NAD^+^ ratios of *Synechocystis* 6803 GT, *Δddh*, *ΔackA*, and CitHox cells under dark, anaerobic conditions. NADH and the total sum of NAD^+^ and NADH levels were quantified using the NAD/NADH Assay Kit-WST. The intracellular NADH/NAD^+^ ratios were calculated from the NADH levels and the total sum of NAD^+^ and NADH within the cells. The data are presented as relative values, with the values of GT set as 1 after three days of dark, anaerobic incubation. Data is presented as means from four biologically independent experiments. These results represent means from four biologically independent experiments. Asterisks indicate statistically significant differences in the ratios between GT and the mutant strain (Student’s *t*-test; *p < 0.05, **p < 0.005).

In *Δddh*, *ΔackA*, CitHox, and *ΔcitH*, hydrogen production and/or the sum of four-carbon dicarboxylic acids and lactate altered. To determine whether changes in the utilization of pathways involving NADH oxidation resulted in differences the percentage of oxidized forms, intracellular NADH levels and the sum of NAD^+^ and NADH were measured for GT, *Δddh*, *Δacs*, *ΔackA*, CitHox, and *ΔcitH*. To determine the intracellular NAD^+^ levels, we subtracted the amount of NADH measured from the total sum of NAD^+^ and NADH. After subjecting the cells to three days of dark, anaerobic culture, we observed that the intracellular NADH/NAD^+^ ratio decreased to 0.39 ± 0.15 by the *ackA* knockout and to 0.70 ± 0.09 by *citH* overexpression, with GT under the same conditions being set at 1 (Figure 5). In contrast, the *ddh* or *citH* knockout did not lead to any significant alteration in the NADH/NAD^+^ ratio (Figures 5 and Supplementary Figure S1b).

## 4 Discussion

In this study, we demonstrated the correlation between organic acid and hydrogen production and NADH/NAD^+^ ratios under dark, anaerobic conditions in *Synechocystis* 6803. Since PEP and pyruvate are competed as carbon intermediates in organic acid production, the perspective of carbon intermediate competition is important, and the regulation of the expression of genes related to organic acid metabolism and metabolomic analysis has been conducted (Osanai et al., 2015; Hasunuma et al., 2016; Hidese et al., 2020). NADH is essential for the catalytic reactions of MDH and DDH, which metabolize carbon intermediates to organic acids, however, there is a lack of references focusing on NADH, and this study focuses on NADH. Since NAD kinase (sll1415, slr0400), which catalyzes NAD^+^ to NADP^+^ conversion, plays an important role in *Synechocystis* 6803 under heterotrophic conditions (Ishikawa et al., 2016, 2021), NADPH may also act as an electron donor. However, NADH-dependent enzymes act in *Synechocystis* 6803 under dark, anaerobic conditions (Katayama et al., 2022), and hence, NADH is a major electron donor under dark, anaerobic conditions in this cyanobacterium.

Under dark, anaerobic conditions, the *ackA* knockout resulted in a 1.7-fold increase in glycogen consumption compared to GT, while the *ddh* or *acs* knockout or *citH* overexpression had no significant impact (Table 1). Also, the interruption of acetate production resulted in a 2.9- and 2.0-fold increase in lactate production and the sum of four-carbon dicarboxylic acids, respectively (Figure 3). Notably, acetate is a prominent fermentation metabolite in *Synechocystis* 6803 under these conditions (Osanai et al., 2015; Iijima et al., 2021), and its biosynthesis is believed to be an AK dependent pathway (Osanai et al., 2015). AK is also involved in ATP generation, and the absence of *ack*A leads to decreased intracellular ATP levels and alterations in carbon flow. The decrease of glycogen levels in the *ackA* knockout compared to GT suggest that an increased flow toward alternative ATP-generating pathways, such as pyruvate kinase (PYK), potentially accelerating the glucose catabolic pathway (Table 1). Indeed, the activation of PYK in *Synechocystis* 6803 is known to enhance sugar catabolic flux (Knowles and Plaxton, 2003). By the *ackA* knockout, the increased demand for ATP generation by PYK may be accompanied by an increased flow toward pyruvate. Increase in glucose catabolism is also accompanied by increased generation of NADH (Figure 2). Also, PEP is a substrate for PYK and a key branching point in organic acids synthesis (Osanai et al., 2015; Hasunuma et al., 2016; Iijima et al., 2021). The observed increase in lactate and four-carbon dicarboxylic acids by the *ackA* knockout compared to GT may be due to altered carbon source allocation and an increased supply of NADH as a substrate for MDH and DDH through glucose catabolic pathway, with the greater percentage increase in lactate potentially linked to ATP generation.

**Figure 6:**
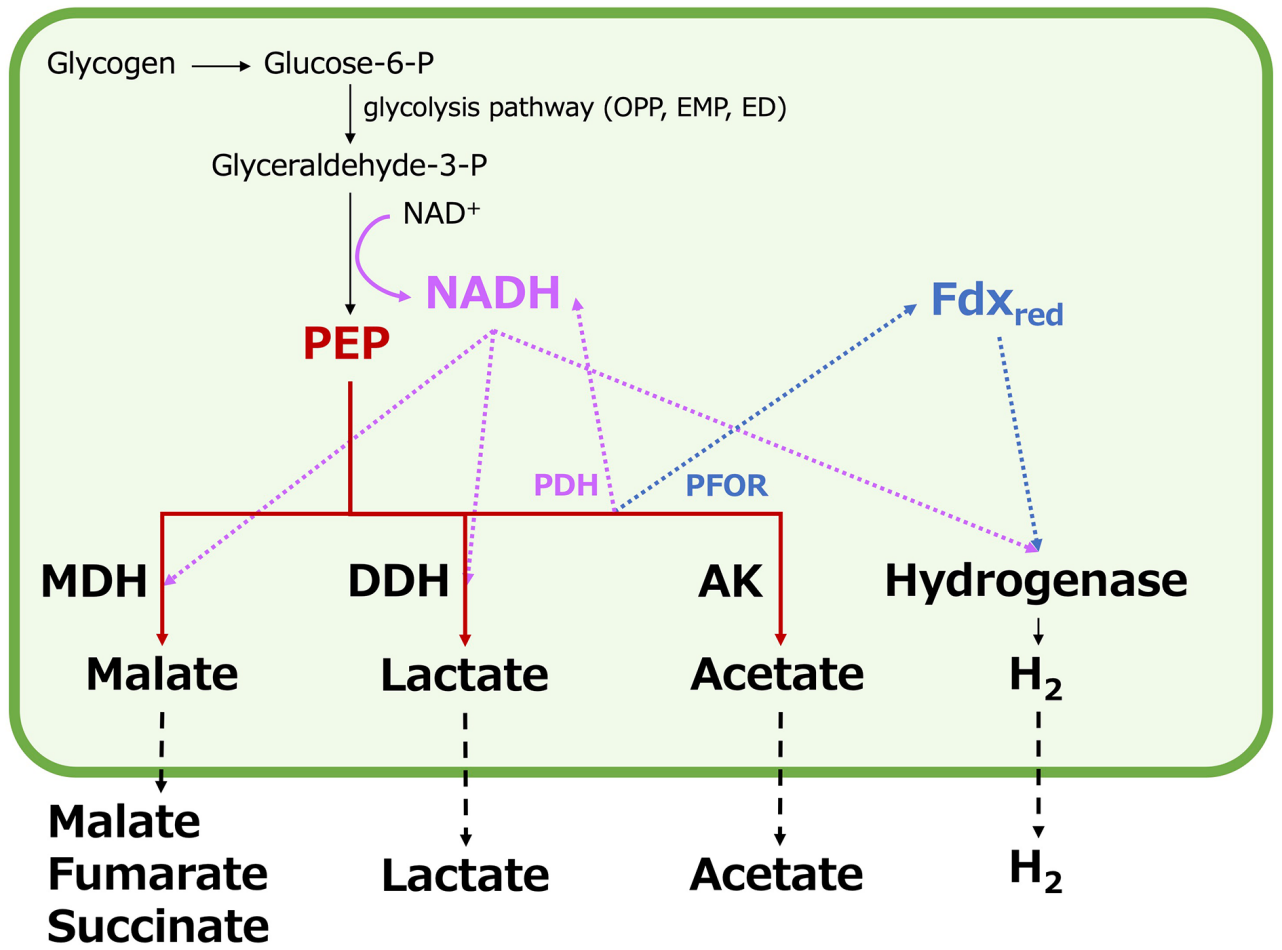
Schematic model of competition for carbon intermediates and reducing power by organic acids and hydrogen production in *Synechocystis* 6803 under dark, anaerobic conditions. P designates phosphate. OPP; oxidative pentose phosphate, EMP; Embden–Meyerhof–Parnas, ED; the Entner–Doudoroff, PEP; phosphoenolpyruvate, MDH; malate dehydrogenase, DDH; d-lactate dehydrogenase, PDH; pyruvate dehydrogenase, PFOR; pyruvate: ferredoxin oxidoreductase, Fdx_red_; reduced ferredoxin, and AK; acetate kinase.

In *Synechocystis* 6803 under dark, anaerobic conditions, the acetate production pathway was found to affect NADH oxidation (Figure 5). Decreased hydrogen production due to the *ackA* knockout indicates decreased consumption of NADH or reduced ferredoxin by hydrogenases (Figure 4). Increased four-carbon dicarboxylic acids and lactate production and decreased NADH/NAD^+^ ratios by the *ackA* knockout are due to increased NADH oxidation by MDH and DDH (Figures 3, 5), explaining the decreased hydrogen production in terms of NADH competition. A decrease in acetate was also observed by the *ackA* knockout (Figure 3). Pyruvate dehydrogenase (PDH) and pyruvate:ferredoxin oxidoreductase (PFOR), which catalyze pyruvate decarboxylation, produce NADH or reduced ferredoxin, respectively and contribute to providing reducing power (Figure 6) (Tittmann, 2009). The decrease in acetate suggests a decrease in flow to the acetate production pathway through PDH and PFOR and a decrease in the supply of reducing power to hydrogenase, reinforcing the reason for the decrease in hydrogen production.

The low NADH/NAD^+^ ratio by *citH* overexpression suggests increased NADH oxidation by MDH (Figure 5). Compared to GT, overexpression of *citH* resulted in a 2.6-, 0.3-, and 0.4-fold increase in four-carbon dicarboxylic acids, lactate, and hydrogen production, respectively (Figures 3, 4). The increase in four-carbon dicarboxylic acids due to *citH* overexpression is consistent with previous results (Iijima et al., 2021). The decrease in lactate may result from competition for NADH and the carbon source between MDH and DDH, while the decrease in hydrogen may result from competition for NADH between MDH and hydrogenase (Figure 6). The overall increase in total organic acids production by 25% by the *citH* overexpression compared to GT (Figure 4) may be attributed to a concurrent increase in carbon dioxide assimilation by phosphoenolpyruvate carboxylase (PEPC), an enzyme acting just before MDH.

The *ddh* knockout did not lead to an increase in the production of four-carbon dicarboxylic acids and hydrogen (Figures 3, 4). Also, acetate production, which supplies reducing power, did not increase by *ddh* knockout, and the NADH/NAD^+^ ratio was maintained at the same level as in GT (Figures 3, 5), suggesting that the *ddh* knockout has a limited effect on the production of fermentation products with oxidation of reducing power by MDH or hydrogenase. Meanwhile, DDH has the capacity to utilize oxaloacetate as a substrate, albeit with only approximately 20% of the activity for pyruvate (Ito et al., 2017). This suggests a potential involvement of DDH in succinate production. Thus, it is possible the production of four-carbon dicarboxylic acids by MDH was not increased in appearance by the *ddh* knockout.

The *citH* knockout resulted in a 1.4-fold increase in hydrogen production compared to GT (Supplementary Figure S1a). Notably, the catalytic efficiency (*k_cat_*/*K*_m_) for NADH analyzed at close pH and temperature differs between MDH and DDH, with MDH exhibiting a higher value of 1512 ± 274 s^−1^ mM^−1^ (pH 7.8, 30°C) compared to DDH’s 94.33 ± 7.83 s^−1^ mM^−1^ (pH 7.5, 30°C) (Ito et al., 2017; Katayama et al., 2022). This observation highlights that MDH is more efficient in catalyzing NADH oxidation than DDH, particularly in the presence of sufficient NADH. The results from our study support the notion that MDH plays a more significant role in NADH oxidation compared to DDH, in line with biochemical analysis.

Our study has demonstrated that the intracellular redox balance of *Synechocystis* 6803, particularly the NADH/NAD^+^ ratio, correlates with the production of organic acids and hydrogen under dark, anaerobic conditions. Specifically, if the NADH/NAD^+^ ratio value of the mutant strain is lower than that of GT, production of four-carbon dicarboxylic acids and lactate is likely to occur, while hydrogen production is unfavorable. This finding provides a novel perspective on organic acids and hydrogen production under dark, anaerobic conditions by *Synechocystis* 6803. In estimating the amount of extracellular metabolites, the measurement of the NADH/NAD^+^ ratio was useful and decreased the number of parameters to be measured. However, it remains elucidated whether the changes in NADH/NAD^+^ ratio is caused by the altered production of fermentation products or the changes in NADH/NAD^+^ ratio altered the production of fermentation products. Further experiments are needed to clarify these correlations and causal relationships.

## Data availability statement

The original contributions presented in the study are included in the article/Supplementary material, further inquiries can be directed to the corresponding author.

## Author contributions

MA: Data curation, Formal analysis, Investigation, Writing – original draft. TO: Data curation, Funding acquisition, Writing – original draft.
