# Supplementary material for: Regulation of organic acid and hydrogen production by NADH/NAD+ ratio in Synechocystis sp. PCC 6803
Source: Front Microbiol. 2024 Jan 5;14:1332449. doi: 10.3389/fmicb.2023.1332449 (PMC10797119; doi:10.3389/fmicb.2023.1332449)
Supplement: Supplementary file 1 [file Image_1.pdf]

Fig. S1

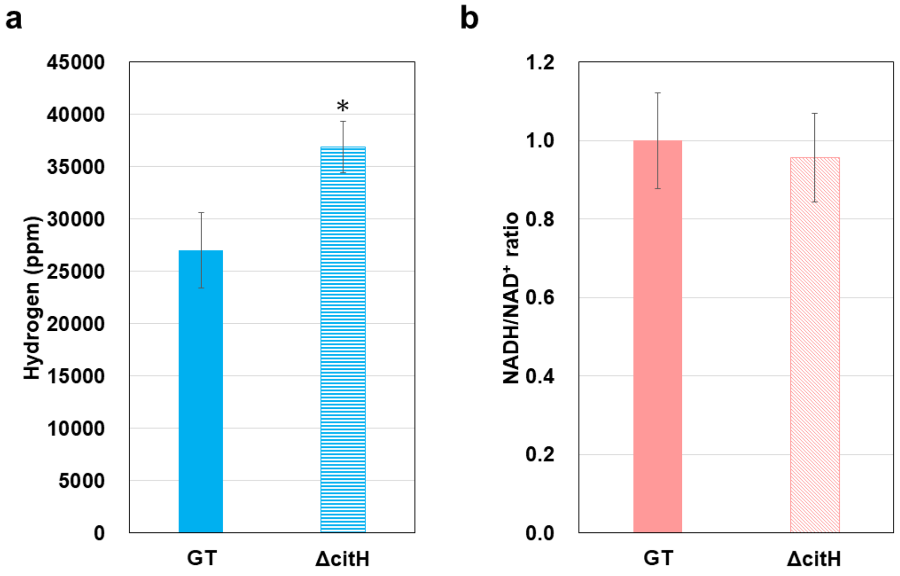

Supplementary Figure 1.

(a) Quantification of hydrogen levels in *Synechocystis* 6803 GT and *ΔcitH* cells under dark, anaerobic conditions. The concentration of hydrogen after three days of incubation under dark, anaerobic conditions was determined using gas chromatography with a thermal conductivity detector. Data is presented as means ± SD from six to eight biologically independent experiments. Asterisk indicates statistically significant difference between GT and the mutant strain (Student's *t*-test; *$P < 0.05$). (b) Ratios of intracellular $NADH/NAD^+$ of *Synechocystis* 6803 GT and *ΔcitH* cells under dark, anaerobic conditions. Intracellular $NADH/NAD^+$ ratios were calculated from the NADH and the total sum of NAD^+ and NADH in cells. The data are presented as relative values, with the values of GT set as 1 after three days of dark, anaerobic incubation. Data is presented as means ± SD from four biologically independent experiments.
